# A Global Longitudinal Study Examining Social Restrictions Severity on Loneliness, Social Anxiety, and Depression

**DOI:** 10.3389/fpsyt.2022.818030

**Published:** 2022-03-28

**Authors:** Michelle H. Lim, Pamela Qualter, Lily Thurston, Robert Eres, Alexandra Hennessey, Julianne Holt-Lunstad, Gavin W. Lambert

**Affiliations:** ^1^Iverson Health Innovation Research Institute, Swinburne University of Technology, Hawthorn, VIC, Australia; ^2^Centre for Mental Health, Swinburne University of Technology, Hawthorn, VIC, Australia; ^3^Manchester Institute of Education, University of Manchester, Manchester, United Kingdom; ^4^Orygen Research Centre, Parkville, VIC, Australia; ^5^Murdoch Children Research Institute, Centre of Research Excellence: CP-Achieve, Neurodisability and Rehabilitation, Melbourne, VIC, Australia; ^6^Department of Paediatrics, The University of Melbourne, Parkville, VIC, Australia; ^7^Department of Psychology, Brigham Young University, Provo, UT, United States

**Keywords:** SARS-CoV-2, social restrictions, loneliness, depression, social anxiety

## Abstract

**Purpose:**

Social restrictions and government-mandated lockdowns implemented worldwide to kerb the SARS-CoV-2 virus disrupted our social interactions, behaviours, and routines. While many studies have examined how the pandemic influenced loneliness and poor mental health, such as depression, almost none have focussed on social anxiety. Further, how the change in social restrictions affected change in mental-health and well-being has not yet been explored.

**Methods:**

This is a longitudinal cohort study in community dwellers who were surveyed across three timepoints in the first six months of the pandemic. We measured loneliness, social anxiety, depression, and social restrictions severity that were objectively coded in a sample from Australia, United States, and United Kingdom (*n* = 1562) at each time point. Longitudinal data were analysed using a multivariate latent growth curve model.

**Results:**

Loneliness reduced, depression marginally reduced, and social anxiety symptoms increased as social restrictions eased. Specific demographic factors (e.g., younger age, unemployment, lower wealth, and living alone) all influenced loneliness, depression, and social anxiety at baseline. No demographic factors influenced changes for loneliness; we found that those aged over 25 years reduced faster on depression, while those younger than 25 years and unemployed increased faster on social anxiety over time.

**Conclusion:**

We found evidence that easing social restrictions brought about additional burden to people who experienced higher social anxiety symptoms. As country-mandated lockdown and social restrictions eased, people are more likely report higher social anxiety as they readjust into their social environment. Mental health practitioners are likely to see higher levels of social anxiety in vulnerable communities even as social restrictions ease.

## Introduction

Efforts to reduce the spread of the severe acute respiratory syndrome coronavirus 2 (SARS-CoV-2) have led to the implementation of local, national, and international public health restrictions (1). Central to these restrictions is reducing social interactions including social distancing, quarantine, and self-isolation (2). Such public health restrictions simultaneously pose barriers in initiating and maintaining social relationships and interactions (3). This could lead to increased loneliness, an unpleasant feeling that arises when one feels one’s actual level of social connection does not meet one’s desired level of connection (4). Before the public health crisis, loneliness was recognised as an emerging public health issue (5), with robust evidence indicating negative implications for physical and mental health (6, 7) across the lifespan (8).

In a nationally representative United Kingdom study, loneliness was reported to be stable over the first 7-week lockdown period, except for those who were categorised in the highest or lowest loneliness groups (9). Those in the highest loneliness group at the beginning of the lockdown experienced increased loneliness and those in the lowest loneliness group experienced a decrease in loneliness before rebounding to their starting level by week six of the lockdown period (9). Other studies have shown age-dependent divergence—with decreases in loneliness among younger adults and increases in loneliness among older adults during lockdown periods in the United Kingdom and United States (10, 11).

A meta-analysis that examined the psychological impact of lockdowns on mental health found small but significant impacts on anxiety and depression but not on loneliness, general distress, and positive psychological functioning (12). However, the meta-analysis reported heterogeneity across studies, reflecting the difficulty of studying lockdowns across countries and at different time points across the pandemic (12). Crucially, none of the studies included in the meta-analysis examined the impact of the *severity of social restrictions* on loneliness and mental health.

The study of how social restrictions affected reports of loneliness or mental health are also more likely to use cross-sectional design. In cross-sectional studies, stay-home orders contributed to higher depression and loneliness in the United States (13) and were associated with higher anxiety, depression, and loneliness in Germany (14). Another distinct gap in the current literature on mental health during the SARS-CoV-2 pandemic was the inclusion of social anxiety symptoms. It is plausible to expect social anxiety may ease due to reduced social interactions or increased due to changes in social routines. Furthermore, social anxiety is highly related to loneliness and depression in the general community (15).

The SARS-CoV-2 lockdowns provided conditions of a natural experiment to explore how social restrictions influenced loneliness, depression, and social anxiety, and the relationships between them. We examined changes in loneliness, depression, and social anxiety, identifying specific demographic differences that affected initial experience and rate of change. Given the variation over the first 6 months in the severity of social restrictions imposed, we also explored whether decreasing social restrictions influenced the rate of change in loneliness, depression, and social anxiety.

## Methods

### Participants

**FIGURE 1:**
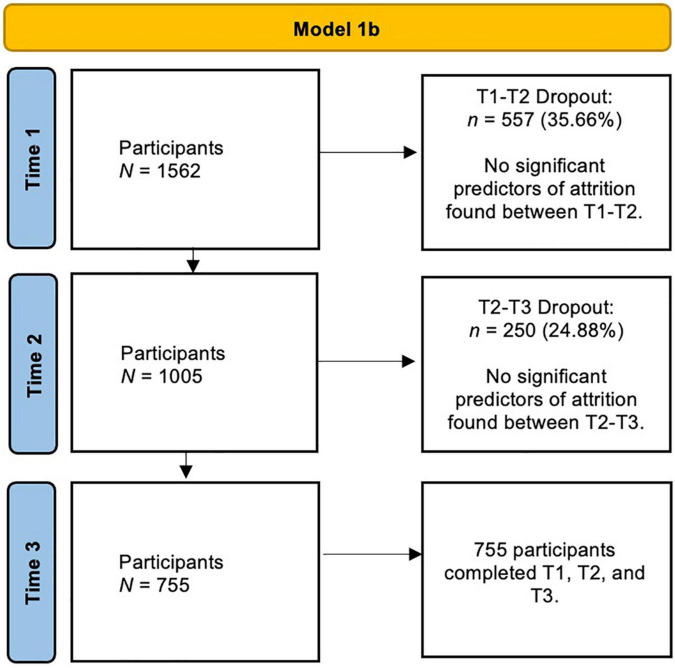
Flowchart for participants in Model 1b. Multivariate latent growth curve model (MLGC) accounts for missing data, and those who had a subsequent timepoint are considered completers.

A total of 2,665 participants across 121 countries completed questionnaires at three time points during the first 6 months of the SARS-CoV-2 pandemic. We recruited participants from organisations interested in monitoring the impact of COVID on loneliness and mental health and the general public. In the current paper, we restrict our analyses to data collected from participants residing in three countries [*N* = 1,562: Australia (*n* = 701), United Kingdom (*n* = 483), and United States (*n* = 378)] because we could reliably extract and code social restriction severity data at the three time points of data collection. Figure 1 shows the participant recruitment flow chart across time, including dropout rates at each of the three time points; we found no demographic differences (*ps* > 0.05) between those who dropped out (i.e., those who only did T1) versus non-dropouts (i.e., T1–T2, T1–T2–T3 completers^1^). Table 1 presents demographic information for the entire sample and the subsample whose data were used in the current study.

**TABLE 1:** Demographics across entire sample and subsample.

Item	Full sample	Subsample
	2,665	1,562
**Gender *n* (%)**		
Male	444 (17%)	217 (13.9%)
Female	2,169 (83%)	1,315 (84.2%)
Intersex	1 (<1%)	1 (<1%)
Transgender	12 (<1%)	8 (<1%)
Other	27 (1%)	16 (1%)
Prefer not to say	10 (<1%)	4 (<1%)
**Age**		
Mean age	47.62	48.80
Range	18–91	18–91
**Relationship status *n* (%)**		
In a relationship/married	1,585 (61%)	974 (62.4%)
Single (including separated/divorced/widowed)	982 (37.8%)	567 (36.3%)
Other	31 (1.2%)	19 (1.1%)
**Work status *n* (%)**		
Full-time	1,232 (47.4%)	684 (43.8%)
Part-time/casual/self-employed	581 (22.4%)	379 (24.3%)
Student	136 (5.2%)	84 (5.4%)
Unemployed	199 (7.7%)	121 (7.7%)
Retired	361 (13.9%)	243 (15.6%)
Other	90 (3.5%)	50 (3.2%)
**Household status *n* (%)**		
Living with family	1,863 (71.7%)	1,107 (70.9%)
Living alone	561 (21.6%)	355 (22.7%)
Other, i.e., living with non-family members	173 (6.6%)	98 (6.3%)
**Financial status *n* (%)**		
Very well	958 (36.9%)	639 (41%)
Fairly well	1,297 (50%)	760 (48.7%)
Poorly	340 (13.1%)	160 (10.2%)
**Education level *n* (%)**		
High school	516 (19.9%)	342 (21.9%)
Bachelor’s degree	991 (38.2%)	573 (36.7%)
Postgraduate degree (i.e., Master’s, Doctorate)	1,087 (41.9%)	643 (41.3%)
**SARS-CoV-R exposure**		
Total contact with COVID-19 (reported knowing others who have had COVID-19 [friends, family, or co-worker])	158 (5.9%)	62 (4.0%)
Has had COVID-19 (reported having symptoms of COVID and/or a positive test result).	405 (15.2%)	168 (10.9%)

*The subsample data comes only from the United Kingdom, United States, and Australia because we were able to calculate objective social restrictions for participants geographic region based on government data across the first six months of the pandemic. For analyses, we removed data from 29 participants who selected “Other,” “Intersex,” “Transgender,” or “Prefer not to say” for their gender because they represented one or less percent of the total group. Raw values may not add up to total because of missing data on those items. Percentages calculated using raw score and total score for that particular item. Total contact with COVID-19 (0 = know others with COVID-19 (friends, family, and co-workers), 1 = does not know anyone with COVID-19; Has had COVID-19 (0 = has had a positive test result for COVID-19 or has had symptoms, 1 = no symptoms or positive test result for COVID-19). Total contact and has had COVID-19 were used as control variables in the analyses.*

### Measures

#### Demographic Form

Demographic data relating to the age, gender, relationship status, work status, financial status, household status, and whether the individual was a carer or parent, education level, and postcode/zipcode were collected (see Table 1). For analyses, we recoded data as follows: age (0 = 18–25^2^ years of age, 1 = 25–65 years of age), gender (1 = female, 2 = male), work status (0 = unemployed, 1 = working full -or part- time), financial status (0 = poor, 1 = fairly well off or well off), household status (0 = living alone, 1 = living with others), carer (0 = yes, 1 = no), and parent of children younger than 16 years (0 = yes, 1 = no). Data for relationship status and education level were significantly skewed and therefore excluded in the analyses. Postcode/zipcode data were used to create data on social restrictions, as noted below.

#### Loneliness. UCLA Loneliness Scale – Version 3

The UCLA Loneliness Scale – Version 3 (UCLA-LS; 16) is a 20-item measure employing a 1 (Never) to 4 (Always) Likert scale, assess loneliness severity. The UCLA-LS has previously been found to demonstrate good to excellent reliability (α = 0.89–0.94) and acceptable test-retest reliability (*r* = 0.73; 16). In the current sample, the UCLA-LS has excellent internal consistency across timepoints (αs = 0.94–0.95).

#### Depression. Patient Health Questionnaire-8

The Patient Health Questionnaire-8 (PHQ-8; 17) is an 8-item measure of depression severity based on DSM-IV criteria. The PHQ-8 has demonstrated sensitivity of 99%, specificity of 92%, and a positive predictive value of 57% when using a cutoff score of 10 or more (17). In the current sample, the PHQ-8 had excellent internal consistency across timepoints (αs = 0.89–0.90).

#### Social Anxiety. Mini-Social Phobia Inventory

The Mini-Social Phobia Inventory (Mini-SPIN; 18) is a 3-item measure of generalised social anxiety disorder, using a 5-point Likert scale ranging from 1 (not at all) to 5 (extremely). The Mini-SPIN has demonstrated excellent internal consistency (α = 0.90) and good test-retest reliability (*r* = 0.82) (19). In the current sample, the Mini-SPIN had good internal consistency across timepoints (αs = 0.82–0.85).

#### Social Restrictions Severity

At each time point of data collection, we coded the number of social restrictions implemented in the United Kingdom, Australia, and United States, based on a variety of government-sanctioned guidelines that mirrored the subjective social restrictions (e.g., border, school, restaurant closures). See Supplementary Tables 1–6 for details. Independent coders received training on agreed guidelines for coding social restrictions based on information about restrictions from each location. Two authors (LT and RE) were randomly allocated 10% of the data and intra-class correlations confirmed reliability between each of the three coders’ scoring within each country (*r* values of 0.75, 0.83, and 0.95, for the United Kingdom, United States, and Australia, respectively).

We then generated a social restriction severity variable for each time point of data collection to examine how the severity of social restrictions changed over time, and influenced the variables of interest. We created a restriction score by completing three steps. First, an objective restriction score was created. Objective restrictions were measured on a dichotomous scale with 0 = restriction not in place and 1 = restriction in place based on the current governmental advice for each person based on their geographical location. Scores were summed together and divided by the total number of possible restrictions (i.e., 12 total restrictions). Second, a restriction severity was created. Restriction severity was measured on a scale anywhere from 0 (no restriction) to 5 (most severe restriction) depending on the variable being coded. See Supplementary Table 1 for severity coding range for different social restrictions. Scores were summed together and divided by the total possible severity score (i.e., a severity score of 36). Finally, to ensure that we accounted for the number of restrictions impacting the severity scores, we multiplied the objective restrictions by severity (represented as objective restriction score × restriction severity score).

#### SARS-CoV-2 Exposure

We assessed whether participants had a current or previous diagnosis of COVID-19 because this could confound results. Response options included, “Yes I suspect I have (or have previously had) COVID-19 but no formal test was taken,” “Yes I have (or had) COVID-19 which was diagnosed through a positive test result,” or “No, I do not have (or have not had) COVID-19.” Participants were also asked if they knew anyone who had tested positive for the virus within the last 14 days and, if yes, whether they had been in close contact with that person. Participants provided this information at each time point of data collection. Using those data, we created two new variables that we included in our analyses as control variables: (1) total contact with COVID-19 [0 = know others with COVID-19 (friends, family, co-workers), 1 = does not know anyone with COVID-19] and (2) has had COVID-19 (0—has had a positive test result for COVID-19 or has had symptoms, 1 = no symptoms or positive test result for COVID-19).

### Procedure

Ethics approval was granted by the Swinburne University of Technology Human Research Ethics Committee. Participants were recruited via collaborative organisation networks, media, and digital advertising and gave consent online. We administered three online surveys across three time points (T1, T2, and T3) where each time point was 6–8 weeks apart, beginning March 2020. Participation was voluntary. See Figure 1.

### Data Analysis Plan

Longitudinal data on loneliness, depression, social anxiety, and social restrictions were analysed using a multivariate latent growth curve model (MLGC) in Mplus (20). Our MLGC model is a single model of growth in loneliness, depression, social anxiety, and social restrictions where we fit the four simultaneous growth curves and estimate covariances among their growth factors. We used linear growth models with continuous outcomes; models were estimated using the robust maximum likelihood (MLR) estimator, to account for missing data (21). In addition, (a) the coefficients for each intercept factor were fixed to zero, (b) the intercepts were fixed to zero, (c) the means and variances of both the intercept and slope factors were estimated, (d) the factor co-variances between each slope-intercept pair were estimated, (e) cross-domain factor covariances were estimated, (f) residual variances were estimated and allowed to vary across time points, and (g) residual covariances were assumed to be zero.

Model fit was evaluated using RMSEA, CFI, TLI, and SRMR. RMSEA values of less than 0.05 indicate a close fit, and values up to 0.08 represent reasonable errors of approximation, and TLI and CFI values ≥0.95 represent good fit (22); a cut-off value of <0.09 for the SRMR (23). Variances in the model were also explored to determine whether there was justification to incorporate predictor variables into subsequent analyses to explain the parameter estimates.

In the first model (Models 1a) we (a) explored the growth of loneliness, depression, social anxiety and social restrictions over the first six months of the pandemic, (b) evaluated how the initial severity of social restrictions and the rate of change in social restrictions affected changes in loneliness, depression, and social anxiety, and (c) determined whether change in loneliness, depression, and social anxiety affected change in each other over time. In the second model (Model 1b), we added demographic information into the model to explore whether individual differences predicted change in loneliness, depression, and social anxiety over time, while controlling for severity of initial social restrictions and the change in social restrictions by having those variables in the model. Our data met the criteria for using MLGC, including having a minimum sample size of at least 200 participants at each time point (24). We used *p* < 0.05.

We conducted two sensitivity analyses as follows: (1) exploration of the growth of loneliness, depression, and social anxiety for the full sample, where social restrictions data were not available for all participants, to determine whether the same patterns of change in loneliness, depression, and social anxiety were observed for the full sample (Model SA1; results found in Supplementary Tables 7, 8) and (2) exploration of the model fit statistics, patterns of change in loneliness, depression, and social anxiety, and the effects of initial and change in social restrictions on loneliness, depression, and social anxiety for participants who had complete data at all three time points (Model SA2; results found in Supplementary Tables 9, 10).

## Results

In Model 1a, baseline and change in loneliness, depression, social anxiety and secerity of social restrictions did not fit the data particularly well (RMSEA = 0.076 [0.073, 0.078], CFI = 0.790, TLI = 0.752, SRMR = 0.085). Adding the predictors to the model (Model 1b) provided a much better model fit (RMSEA = 0.057 [0.054, 0.061], CFI = 0.928 TLI = 0.881 SRMR = 0.045).

The most variability in the model was in severity of restrictions at six months into the pandemic (covariance = 30.05 at T3 compared to 15.18 at T1 and 6.83 at T2); depression, loneliness, and anxiety also showed the most variability at six months (T3; see Table 2 under covariances). Table 2 also shows that the strongest associations were between loneliness and depression at T3 (0.61), loneliness and social anxiety at T3 (0.50), and depression and social anxiety at T2 (0.50). All correlations between T1 and T2 variables were *rs*≥0.425, and T2 and T3 variables were *rs* ≥0.437, *p* < 0.001. Correlations between social restriction severity and loneliness, depression, and social anxiety at each time point was always small (*rs* < −0.09).

**TABLE 2:** Estimated sample statistics for Model 1b: covariances, correlations, and change in loneliness, depression, social anxiety, and SARS-CoV-2 social restrictions.

	Estimated sample statistics													
	
	Means	Loneliness				Depression			Social anxiety			Restrictions severity		

		**T1**		T2	T3	T1	T2	T3	T1	T2	T3	T1	T2	T3
		45.76		46.00	45.91	8.38	7.82	8.02	3.72	3.75	4.03	23.09	19.47	18.66

	**Covariances**	**Loneliness**				**Depression**			**Social anxiety**			**Restrictions severity**		
					
		**T1**		**T2**	**T3**	**T1**	**T2**	**T3**	**T1**	**T2**	**T3**	**T1**	**T2**	**T3**

Loneliness	T1	125.05												
	T2	111.01		136.78										
	T3	112.60		123.21	143.93									
Depression	T1	36.39		36.31	37.43	35.42								
	T2	33.43		40.91	39.42	26.05	33.76							
	T3	33.92		38.20	43.40	25.62	26.97	35.32						
Social anxiety	T1	17.04		16.52	16.66	8.95	7.51	7.56	10.10					
	T2	14.97		17.40	17.55	8.11	9.02	8.37	7.22	9.90				
	T3	17.34		19.09	20.01	8.75	8.12	9.75	7.75	7.75	11.01			
Restrictions severity	T1	−2.18		–2.46	–1.72	0.02	–1.15	–0.63	–0.58	–0.39	–1.10	15.18		
	T2	−0.84		–0.79	–1.54	–0.73	–1.03	–0.82	–0.33	–0.48	–0.77	5.77	6.83	
	T3	−2.69		–2.80	–2.69	–0.02	–1.40	.43	–0.14	.20	–0.22	13.74	8.87	30.05

	**Correlations**													
	
		**Loneliness**				**Depression**			**Social anxiety**			**Restrictions severity**		
					
		**T1**		**T2**	**T3**	**T1**	**T2**	**T3**	**T1**	**T2**	**T3**	**T1**	**T2**	**T3**

Loneliness	T1	1.00												
	T2	0.85		1.00										
	T3	0.84		0.88	1.00									
Depression	T1	0.55		0.52	0.52	1.00								
	T2	0.52		0.60	0.57	0.75	1.00							
	T3	0.51		0.55	0.61	0.72	0.78	1.00						
Social anxiety	T1	0.48		0.45	0.44	0.47	0.41	0.40	1.00					
	T2	0.43		0.47	0.47	0.43	0.49	0.45	0.72	1.00				
	T3	0.47		0.49	0.50	0.44	0.46	0.50	0.74	0.74	1.00			
Restriction severity	T1	−0.05		–0.05	0.04	0.00	–0.05	–0.03	–0.05	–0.03	–0.09	1.00		
	T2	−0.03		–0.03	0.05	–0.05	–0.07	–0.05	–0.04	–0.05	–0.09	0.57	1.00	
	T3	−0.04		–0.04	0.04	0.00	–0.04	0.01	–0.01	0.01	–0.01	0.64	0.62	1.00

	**Model results**													
	
			**Estimate**			**Standard error (SE)**			**Estimate/SE**			***p*** **-value**		

	Intercept loneliness		46.04			0.22			208.31			<0.001		
	Slope loneliness		−0.47			0.03			−14.33			<0.001		
	Intercept depression		8.31			0.12			72.45			<0.001		
	Slope depression		−0.09			0.02			−4.20			<0.001		
	Intercept social anxiety		3.44			0.06			60.44			<0.001		
	Slope social anxiety		0.65			0.01			47.56			<0.001		
	Intercept social restrictions		22.38			0.09			254.82			<0.001		
	Slope social restrictions		−0.92			0.02			−38.49			<0.001		

*Latent growth curve model (LGCM) includes data from the subsample whose country level data on social restristrictions during the first six months of the COVID-19 pandemic could be retrieved (N = 1,562). Linear growth models were estimated, with continuous outcomes; models were estimated using the robust maximum likelihood (MLR) estimator, to account for missing data (20).*

Exploration of the intercepts showed social restrictions to be high across the sample at baseline; loneliness and social anxiety were relatively low, comparable to pre-COVID data (25), but depression was slightly higher (26). Examination of the estimates for the slopes (see Table 2) showed a significant reduction in loneliness over the first six months of the pandemic (−0.47), a significant, but small change in depression over time (0.09), an increase in social anxiety (0.65), and a reduction in social restrictions (−0.92).

Table 3 shows where a person started on loneliness did not predict change in loneliness and where a person started on depression did not predict change in depression, but where they started on social anxiety did predict change in social anxiety: those higher on social anxiety at baseline (T1) had a faster rate of change in social anxiety throughout the pandemic, such that those higher on social anxiety at baseline increased on social anxiety faster people who scored lower at baseline. Where people started on depression and social anxiety predicted change in loneliness over the course of the project: people higher on depression or social anxiety reduced slower on loneliness. In addition, the rate of change in social restrictions affected the rate of change in social anxiety, with levels of social anxiety increasing fastest where restrictions were easing (reducing) fastest.

**TABLE 3:** Parameter estimates for Model 1b: predicting associations between change in loneliness, depression, social anxiety, and SARS-CoV-2 social restrictions.

Model results				

	Estimate	Standard error (SE)	Estimate/ *SE*	*p*-value
Intercept of loneliness → slope of loneliness	–0.14	0.08	–1.85	0.07
**Intercept of depression →**				
Slope of depression	–0.07	0.12	–0.60	0.55
Intercept of loneliness	0.66	0.02	29.43	<0.001
Slope of loneliness	–0.31	0.10	–3.02	0.003
**Intercept of social anxiety →**				
Slope of social anxiety	1.68	0.36	4.62	<0.001
Intercept of loneliness	0.60	0.02	28.42	<0.001
Slope of loneliness	–0.54	0.15	–3.70	<0.001
Intercept for depression	0.61	0.02	25.58	<0.001
Slope of depression	–0.09	0.08	–1.16	0.25
**Slope of depression →**				
Intercept of loneliness	–0.12	0.07	–1.58	0.12
Slope of loneliness	1.50	0.62	2.41	0.02
**Slope of social anxiety →**				
Intercept of loneliness	0.92	0.18	5.03	<0.001
Slope of loneliness	–0.58	0.23	–2.48	0.01
Intercept of depression	0.85	0.17	5.07	<0.001
Slope of depression	0.43	0.21	2.08	0.04
**Intercept of social restrictions →**				
Slope of social restrictions	3.06	0.21	14.94	<0.001
Intercept of loneliness	–0.16	0.11	–1.41	0.16
Slope of loneliness	–0.02	0.02	–1.13	0.26
Intercept of depression	–0.16	0.07	–2.32	0.02
Slope of depression	0.00	0.01	0.32	0.75
Intercept of social anxiety	–0.05	0.03	–1.57	0.12
Slope of social anxiety	–0.12	0.01	–2.08	0.04
**Slope of social restrictions →**				
Intercept of loneliness	–0.50	0.37	–1.35	0.18
Slope of loneliness	–0.03	0.05	–0.58	0.57
Intercept of depression	–0.21	0.21	–1.00	0.32
Slope of depression	–0.04	0.04	–1.16	0.25
Intercept of social anxiety	–0.05	0.03	–1.58	0.12
Slope of social anxiety	–0.07	0.02	–2.84	0.01

*LGCM includes data from the subsample whose country level data on social restristrictions during the first six months of the COVID-19 pandemic could be retrieved (N = 1562). Linear growth models were estimated, with continuous outcomes; models were estimated using the robust maximum likelihood (MLR) estimator, to account for missing data (20).*

Our model results showed that the following were significant predictors of loneliness at baseline (Table 4): being younger (18–25 years), being a carer, being a parent, being unemployed, having lower than average wealth, and living alone. Infact, of all our predictor variables, it was only gender that did not predict loneliness. The following variables significantly predicted depression and social anxiety at baseline (T1): being in the 18–25 year age group, lower than average wealth, and being unemployed.

**TABLE 4:** Parameter estimates for Model 1b: demographic predictors of change in loneliness, depression, and social anxiety.

Model results				

	Estimate	Standard Error (SE)	Estimate/SE	*p*-value
**Predictors of intercept of loneliness**				
Gender	0.04	0.02	1.71	0.09
Age group (18–25 years)	–0.10	0.02	–4.58	<0.001
Being a carer	–0.06	0.02	–2.63	<0.001
Being a parent	0.05	0.02	2.38	0.02
Wealthy	–0.19	0.02	–8.51	<0.001
Unemployed	–0.14	0.02	–6.69	<0.001
Living alone	–0.16	0.02	–7.66	<0.001
**Predictors of slope of loneliness**				
Gender	0.05	0.06	0.83	0.41
Age group (18–25 years)	0.12	0.07	1.81	0.07
Being a carer	0.02	0.06	0.39	0.72
Being a parent	–0.12	0.07	–1.68	0.09
Wealthy	0.02	0.08	0.25	0.81
Unemployed	0.05	0.06	0.84	0.40
Living alone	0.07	0.06	1.28	0.20
Total contact with COVID-19^1^	0.08	0.12	0.62	0.53
Has had COVID (positive test)^1^	–0.12	0.18	–0.68	0.50
**Predictors of intercept of depression**				
Gender	–0.04	0.02	–1.76	0.08
Age group (18–25 years)	–0.19	0.02	–8.33	<0.001
Being a carer	–0.03	0.02	–1.51	0.13
Being a parent	0.01	0.02	0.38	0.71
Wealthy	–0.24	0.02	–10.29	<0.001
Unemployed	–0.12	0.02	–5.03	<0.001
Living alone	–0.04	0.02	–1.77	0.08
**Predictors of slope of depression**				
Gender	0.11	0.07	1.54	0.12
Age group (18–25 years)	0.20	0.10	2.08	0.04
Being a carer	–0.02	0.07	–0.34	0.73
Being a parent	–0.03	0.07	–0.36	0.72
Wealthy	–0.02	0.08	–0.19	0.85
Unemployed	–0.02	0.07	–0.22	0.83
Living alone	0.02	0.06	0.27	0.79
Total contact with COVID-19^1^	0.01	0.13	0.06	0.96
Has had COVID^1^	–0.02	0.18	–0.13	0.90
**Predictors of intercept of social anxiety**				
Gender	–0.02	0.02	–0.67	0.50
Age group (18–25 years)	–0.14	0.03	–5.81	<0.001
Being a carer	–0.04	0.02	–1.85	0.07
Being a parent	0.03	0.02	1.09	0.28
Wealthy	–0.12	0.03	–4.78	<0.001
Unemployed	–0.14	0.03	–5.39	<0.001
Living alone	0.01	0.02	0.59	0.56
**Predictors of slope of social anxiety**				
Gender	0.02	0.04	0.46	0.65
Age group (18–25 years)	–0.20	0.06	–3.42	0.001
Being a carer	–0.04	0.05	–0.87	0.38
Being a parent	0.00	0.04	–0.02	0.99
Wealthy	–0.15	0.05	–2.79	0.01
Unemployed	–0.17	0.05	–3.12	0.002
Living alone	–0.01	0.04	–0.13	0.90
Total contact with COVID-19^1^	–0.01	0.18	–0.06	0.95
Has had COVID^1^	0.04	0.27	0.15	0.88

*^1^Total contact with COVID-19 (0 = know others with COVID-19 (friends, family, and co-workers), 1 = does not know anyone with COVID-19; has had COVID-19 (0 = has had a positive test result for COVID-19 or has had symptoms, 1 = no symptoms or positive test result for COVID-19); both variables used as control variables in the LGCM. LGCM includes data from the subsample whose country level data on social restristrictions during the first six months of the COVID-19 pandemic could be retrieved (N = 1,562). Linear growth models were estimated, with continuous outcomes; models were estimated using the robust maximum likelihood (MLR) estimator, to account for missing data (20).*

Age influenced the rate of change in depression and social anxiety: those aged 18–25 years were slower to reduce on depression, and faster to increase on social anxiety compared to adults older than 25 years. The rate of change in social anxiety was additionally predicted by lower wealth and unemployment: those individuals who had lower perceived wealth and were unemployed increased faster on social anxiety over the first 6 months of the pandemic than those who had more wealth and were employed. None of the variables predicted change in loneliness, suggesting that the rate of change across T1–T3 was negligible between participants.

Sensitivity analyses (see Supplementary Tables 7, 8) showed that the MLGC model for the full sample of data from participants who completed the survey (Model SA1), where the single model included the growth in loneliness, depression, and social anxiety, but did not include social restriction data because those were not available for all countries, was a good fit to the data (RMSEA = 0.064 [0.058, 0.070], CFI = 0.948, TLI = 0.880, SRMR = 0.028). As with our analyses with the smaller subsample, exploration of the intercepts and slopes showed small, but significant reductions in loneliness and depression, and a small increase in social anxiety over six months (see Supplementary Table 7). Further, the same associations between loneliness, depression, and social anxiety were observed, and the same predictors of each were observed with these data as was found for the subsample where the effects of social restrictions could also be included in the model (see Supplementary Table 8).

Further sensitivity analyses (Model SA2) using data from participants who had complete data was also conducted and showed that it was appropriate to use robust maximum likelihood (MLR) estimator to account for such a large amount of missing data from T1 to T3. Indeed, we found the same overall effects using just data for those with data at all three time points (Supplementary Tables 9, 10) as we did for our full subsample where missing data were accounted for using MLR.

## Discussion

The social restrictions mandated to bring the spread of SARS-CoV-2 under control prior to this study had not been examined as predictors of change in individual well-being in the first months of the pandemic. Even though many studies explored the change in depression, loneliness, they did not include in their analyses the rate of change in government-initiated restrictions on social interaction. In the current study, we filled this knowledge gap, and showed that social restrictions negatively impacted the course of social anxiety. Specifically, levels of social anxiety increased fastest where restrictions were easing fastest. This is consistent with features of social anxiety symptoms where lack of social exposure can maintain symptom severity (27, 28). The effect of social restriction severity on depressive symptoms was also examined, and our findings showed that changes in social restrictions did not influence the rate at which people reduced on depressive symptoms. With the effects of severity of social restrictions controlled in our model, we found there was a significant reduction in loneliness, a small, but significant reduction in depression, and an increase in social anxiety over the first six months of the pandemic.

Our findings provide information about how changing social restrictions affected loneliness, depression, and social anxiety, but also provide further evidence of the longitudinal relationships between loneliness and mental health symptomatology. Those higher on loneliness at the start of the pandemic were also higher on depression and social anxiety, suggesting that people reporting one of those issues were more likely to report others, consistent with previous studies showing the close relationships between loneliness and mental health symptom severity (15, 29). These findings further demonstrate that the rate of change in loneliness, social anxiety, or depression affects the rate of change of the other constructs, supporting the potential for psychological therapies to effectively reduce both loneliness and mental health symptom severity (30). Previous research in young people with and without a mental disorder have demonstrated that interventions focussed on addressing loneliness also showed a reduction in social anxiety and depression (29), and psychotic symptomatology (31, 32). In the context of the SARS-CoV-2 pandemic, the reduction in loneliness that accompanied the easing of social restrictions did not lead to reductions in depression and was associated with an increase in social anxiety. It is plausible that relationships between mental health symptomatology observed here are due to the nature of our community sample not looking to address mental health symptomatology through an intervention or perhaps be an artefact of the naturalistic but stressful global environment experienced during the pandemic.

Consistent with previous studies during the SARS-CoV-2 lockdowns (9, 13, 14), we also found that being 18–25 years of age, unemployed, lower than average wealth, and living alone, all predicted higher loneliness, depression, and social anxiety at the start of the pandemic. While no demographic differences predicted the rate of change in loneliness, age predicted the rate of change in depression (those older than 25 years reuced faster), and the rate of change in social anxiety was predicted by age and unemployment, with those younger than 25 years and unemployed being *faster* to increase on social anxiety than those older than 25 years, and employed. These findings are consistent with research that social anxiety also tends to disportionately affect younger people (16–29 years) (33) and adversely affects employment due to decreased social functioning (34).

### Study Limitations

We looked at the impact of *easing* social restrictions rather than *imposing* social restrictions as we did not include data collected pre-pandemic. It is plausible that loneliness and poor mental health symptoms increased *before* T1 data were collected and our data do not speak to the impact of longer-term social restrictions on loneliness and mental health. Additionally, our sample was demographically skewed toward more educated and mostly female participants, similar to other online studies (15).

### Research and Clinical Implications

Our findings support existing literature demonstrating associations between loneliness, depression, and social anxiety over time and address a gap in knowledge about how loneliness and mental health symptoms are related prospectively. These findings are novel: they highlight the impact of social restrictions on mental health outcomes, with specific negative consequences on social anxiety. Loneliness has a reciprocal relationship with social anxiety (15). In the current study, reductions in social anxiety did not accompany reductions in loneliness as restrictions eased. Nonetheless, our study reinforces the importance of measuring related mental health symptom severity in studies focussed on understanding loneliness.

Our findings emphasise the critical need to identify, monitor, and actively intervene as communities recover from lockdowns, with particular importance of assisting vulnerable people (i.e., those unemployed, lower wealth, and younger). Mental health practitioners may see slow or little change in social anxiety symptoms in young people and those unemployed, even as communities move toward reduced social restrictions. While there are valid public health concerns prompting restrictions, our findings should serve as a call to action to assist young people, across different services, from youth mental health, youth centres, educational institutions, and employment.

## Conclusion

As social restrictions eased, loneliness reduced, depression marginally reduced, and social anxiety increased in the first six months of the SARS-CoV-2 pandemic. Social anxiety remains an overlooked mental health symptom and may increase as we attempt to reintegrate socially. Young people, those who are unemployed, living alone, and from lower wealth are all vulnerable groups disadvantaged during SARS-CoV-2 pandemic. Finally, those aged 18–25 and those unemployed continue to experience more social anxiety symptoms even after social restrictions were eased.

## Data Availability Statement

The datasets presented in this study can be found in online repositories. The names of the repository/repositories and accession number(s) can be found below: https://osf.io/58zg2/?view_only=118ed3253c944195aa0c25f352b9aab0.

## Ethics Statement

The studies involving human participants were reviewed and approved by Swinburne University Human Research Ethics Committee. The participants provided their consent to participate in this study online.

## Author Contributions

ML led the conceptualisation of the study, was the overall chief investigator with project management duties including ethics, recruitment, and data collection, and contributed to the writing of the manuscript. PQ led the statistical analyses and contributed to the writing of the manuscript. LT assisted with data collection, inter-rater reliability of the social restrictions coding, and assisted with the writing and formatting of the manuscript. RE assisted with data cleaning, stacking, and the social restrictions coding. AH assisted with data analyses and contributed to the project. JH-L assisted with recruitment and contributed to the reviews and writing of manuscript. GL assisted with the conceptualisation of the project and contributed to the reviews and writing of the manuscript. All authors contributed to the article and approved the submitted version.

## Conflict of Interest

The authors declare that the research was conducted in the absence of any commercial or financial relationships that could be construed as a potential conflict of interest.

## Publisher’s Note

All claims expressed in this article are solely those of the authors and do not necessarily represent those of their affiliated organizations, or those of the publisher, the editors and the reviewers. Any product that may be evaluated in this article, or claim that may be made by its manufacturer, is not guaranteed or endorsed by the publisher.

## Footnotes

1 There were no T1–T3 completers.

2 Young adults aged 18–25 are well known to be more vulnerable to loneliness, and therefore analysed in a different group to those over 25 years old [see (7) for review on age vulnerability].
