# Supplementary material for: A Global Longitudinal Study Examining Social Restrictions Severity on Loneliness, Social Anxiety, and Depression
Source: Front Psychiatry. 2022 Mar 28;13:818030. doi: 10.3389/fpsyt.2022.818030 (PMC8995965; doi:10.3389/fpsyt.2022.818030)
Supplement: Supplementary file 1 [file Data_Sheet_1.docx]

**Table S1**

*Social Restriction Severity Coding Excluding Indoor and Outdoor Gatherings for Australia, United Kingdom, and United States*

| Severity Coding | | | | | | |
| --- | --- | --- | --- | --- | --- | --- |
| Social restriction | 0 | 1 | 2 | 3 | 4 | 5 |
| Closure of gyms, shopping centres, restaurants etc. | Businesses are permitted to operate without any COVID-19 related restrictions | Most places open but operate under COVID-19 safe guidelines | Essential business only. Restaurants operate for take-away only | - | - | - |
| International travel ban/restrictions | Full freedom to travel internationally without restriction | Permitted to leave country of residence but are restriction upon entering any other country (even if just one). Restrictions may include compulsory testing, quarantine, or banned access to that country | Not permitted to leave the country of residence without exemption | Not permitted to leave the country of residence for any reason | - | - |
| Domestic travel ban/restrictions | Full freedom to travel nationally without restriction | Permitted to leave state of residence but are restriction upon entering any other state (even if just one). Restrictions may include compulsory testing, quarantine, or banned access to that country | Not permitted to leave the state of residence without exemption | Not permitted to leave the state of residence for any reason | - | - |
| Border closures/restrictions at a state level^1^ | No restrictions on entering the state from another state | Restricted entry of residents from certain states (even if just one). Restrictions may include house quarantine or compulsory COVID-19 test | Restricted entry of residence from certain states (even if just one). Restrictions include hotel quarantine or compulsory COVID-19 test | Restricted entry of residents from all other states. Restrictions include house and hotel quarantine | Banned entry of residents from certain states (even if just one) | Banned entry of residents from any other state |
| Border closures/restrictions at an international level^2^ | No restrictions on internationally arrivals entering the country | Restricted entry of residents from certain countries (even if just one). Restrictions may include house quarantine or compulsory COVID-19 test | Restricted entry of residence from certain countries (even if just one). Restrictions include hotel quarantine or compulsory COVID-19 test | Restricted entry of residents from all other countries. Restrictions include house and hotel quarantine | Banned entry of residents from certain countries (even if just one) | Banned entry of residents from any other country |
| Compulsory quarantine for anyone who has travelled or had close contact with someone with COVID-19 | No restriction in place | Quarantine in legally enforced for any international or domestic travel or for anyone who has been exposed to a positive COVID-19 case | - | - | - | - |
| Compulsory quarantine for entire population | No restriction in place | Residents are not permitted to leave their residential address for any reason other than for an emergency or to receive medical care | - | - | - | - |
| University closures | No restriction in place | Any impact on mode of content delivery for on campus university courses. Impacts include shifts to online learning (e.g., online lectures) | Full closure with remote learning only | - | - | - |
| School closures | No restrictions. All school aged children required to attend school | Any impact on how the curriculum is delivered. Includes staged returns to schools and restrictions to large school gatherings and excursions | Schools are required to shut down temporarily, force early school holidays or open only for children unable to learn remotely (e.g., children of essential workers) | - | - | - |
| Imposed restrictions on when and for what reasons to leave the place of residence | No restriction in place | Only leave residential address for essential reasons | Only permitted to leave residential address for essential reason plus perimeter in place and/or curfews | - | - | - |

*Note*. ^1^For the United Kingdom this was coded at a national level, i.e., not states but each country within the UK thought of as a state. ^2^For the United Kingdom national is considered to be the entire UK

**Table S2**

*Social Restriction Coding for Outdoor Gatherings across Australia, United Kingdom, and United States*

| Severity Coding | | | | | | |
| --- | --- | --- | --- | --- | --- | --- |
|  | 0 | 1 | 2 | 3 | 4 | 5 |
| Australia | No restriction on outdoor gatherings. | No upper limit to outdoor gathering but must obey density limit (i.e., - 4m^2^ or 2m^2^ maximum per person). | 50-100 providing density limit is met^3^ | 11-49 people providing density limit is met^3^ | 5- 10 person providing density limit is met^3^ | Under 5 |
| England | No restriction on outdoor gatherings. | No upper limit to outdoor gathering but must obey density limit (i.e., - 4m^2^ or 2m^2^ maximum per person). | 50-100 providing density limit is met^3^ | 6 people from different households can meet outdoors or up to 30 people if from two households only. | 6 people from different households can meet outdoors or larger than 6 people if from one household | Under 5 |
| Scotland | No restriction on outdoor gatherings. | Density limit (i.e., - 4m^2^ or 2m^2^ maximum per person) but ban of gatherings over 500 persons. | 50-100 providing density limit is met^3^ | Maximum of 15 people from up to 5 different households | Maximum of 6 people from 2 households or 8 people from 3 households | Under 5 |
| Northern Ireland | No restriction on outdoor gatherings. | No upper limit to outdoor gathering but must obey density limit (i.e., - 4m^2^ or 2m^2^ maximum per person). | 50-100 providing density limit is met^3^ | Maximum of 15 people | Maximum of 10 people | Under 5 |
| Wales | No restriction on outdoor gatherings. | No upper limit to outdoor gathering but must obey density limit (i.e., - 4m^2^ or 2m^2^ maximum per person). | 50-100 providing density limit is met^3^ | Maximum of 30 people | Members of 2 households with social distancing permitted outdoors | Under 5 |
| USA | No restriction on outdoor gatherings. | No upper limit to outdoor gathering but must obey density limit (i.e., - 4m^2^ or 2m^2^ maximum per person). | 50-100 providing density limit is met^3^ | 11-49 people providing density limit is met^3^ | 5- 10 person providing density limit is met^3^ | Under 5 |

*Note*. ^3^Where the number of people permitted according to the density limit (one person per 4 square metres) is less than the gathering limit, the lower number applies.

**Table S3**

*Social Restriction Coding for Indoor Gatherings across Australia, United Kingdom, and United States*

| Severity Coding | | | | | | |
| --- | --- | --- | --- | --- | --- | --- |
|  | 0 | 1 | 2 | 3 | 4 | 5 |
| Australia | No restriction on indoor gatherings. | No upper limit to indoor gathering but must obey density limit (i.e., - 4m^2^ or 2m^2^ maximum per person). | 50-100 providing density limit is met^4^ | 11-49 people providing density limit is met^4^ | 5- 10 person providing density limit is met^4^ | Under 5 |
| England | No restriction on indoor gatherings. | No upper limit to indoor gathering but must obey density limit (i.e., - 4m^2^ or 2m^2^ maximum per person). | 50-100 providing density limit is met^4^ | 2 households (support bubble counts as one household) can meet privately up to maximum 30 people | Maximum 6 people | No indoor gatherings permitted |
| Scotland | No restriction on indoor gatherings. | No upper limit to indoor gathering but must obey density limit (i.e., - 4m^2^ or 2m^2^ maximum per person). | 50-100 providing density limit is met^4^ | 11-49 people providing density limit is met^4^ | Advised to meet people from no more than one other household at a time at home up to 6 people. Meeting people from up to 2 other households at a time indoors (i.e., pubs up to 8 person maximum) even if no household visitation. | No indoor gatherings permitted |
| Northern Ireland | No restriction on indoor gatherings. | No upper limit to indoor gathering but must obey density limit (i.e., - 4m^2^ or 2m^2^ maximum per person). | 50-100 providing density limit is met^4^ | 11-49 people providing density limit is met^4^ | 6 people from 2 different households | No indoor gatherings permitted |
| Wales | No restriction on indoor gatherings. | No upper limit to indoor gathering but must obey density limit (i.e., - 4m^2^ or 2m^2^ maximum per person). | 50-100 providing density limit is met^4^ | 4 households are able to create an extended household and visit privately | 2 households able to join as an extended household and visit privately | No indoor gatherings permitted |
| USA | No restriction on indoor gatherings. | No upper limit to indoor gathering but must obey density limit (i.e., - 4m^2^ or 2m^2^ maximum per person). | 50-100 providing density limit is met^4^ | 11-49 people providing density limit is met^4^ | 5- 10 person providing density limit is met^4^ | Under 5 |

*Note*. ^4^Where the number of people permitted according to the density limit (one person per 4 square metres) is less than the gathering limit, the lower number applies.

**Table S4**

*Sources used for Objective Social Restriction Recording across Australia*

| Victoria | |
| --- | --- |
| Source | URL |
| Victorian Gazette | <http://www.gazette.vic.gov.au/gazette/Gazettes2020/GG2020S144.pdf#page=1>  <http://www.gazette.vic.gov.au/gazette/Gazettes2020/GG2020S135.pdf#page=3>  <http://www.gazette.vic.gov.au/gazette/Gazettes2020/GG2020S135.pdf#page=1> |
| DHHS Victoria | <https://www.dhhs.vic.gov.au/coronavirus/updates/202003>  <https://www.vic.gov.au/Coronavirus-COVID-19-Important-information-for-Victorians> (expired)  <https://www.dhhs.vic.gov.au/sites/default/files/documents/202003/Stay%20at%20Home%20Directions%20.pdf> |
| SBS | <https://www.sbs.com.au/language/english/what-you-can-and-cannot-do-as-melbourne-goes-back-into-lockdownww.s> -lockdown |
| ABC | <https://www.abc.net.au/news/2020-05-11/coronavirus-restrictions-in-victoria-premier-daniel-andrews/12228524>  <https://www.abc.net.au/news/2020-05-24/coronavirus-restrictions-ease-in-victoria-daniel-andrews/12280666>  <https://www.abc.net.au/news/2020-07-07/victoria-reimposes-lockdown-as-coronavirus-cases-rise/12429990>  <https://www.abc.net.au/news/2020-03-16/coronavirus-state-of-emergency-declared-in-victoria/12058442>  <https://www.abc.net.au/news/2020-07-12/coronavirus-victoria-lockdown-remote-learning-schools-covid-19/12433122> |
| Victoria Coronavirus Roadmap | <https://www.coronavirus.vic.gov.au/coronavirus-covid-19-restrictions-roadmaps> |
| Aljazeera | <https://www.aljazeera.com/news/2020/6/3/coronavirus-travel-restrictions-border-shutdowns-by-country> |
| Home affairs.gov | <https://web.archive.org/web/20200330015246/https://covid19.homeaffairs.gov.au/travel-restrictions> |
| Education Victoria | <https://education.vic.gov.au/school/Pages/coronavirus-roadmap-schools.aspx> |
| New South Wales | |
| Legislation NSW | <https://www.legislation.nsw.gov.au/information/covid19-legislation/gathering-and-movement>  Public Health (COVID-19 Restrictions on Gathering and Movement) Order (No 2) 2020  Public Health (COVID-19 Restrictions on Gathering and  Movement) Order 2020  Public Health (COVID-19 Restrictions on Gathering and  Movement) Order (No 2) 2020  Public Health (COVID-19 Gatherings) Order (No 2) 2020 [NSW]  Public Health (COVID-19 Restrictions on Gathering and Movement) Order (No 3) 2020 [NSW]  Public Health (COVID-19 Restrictions on Gathering and Movement) Order (No 4) 2020 [NSW]  Public Health (COVID-19 Border Control) Amendment Order 2020  Public Health (COVID-19 Interstate Hotspots) Order 2020 – 2 July 2020 |
| NSW Government | <https://web.archive.org/web/20200927012453/https://www.nsw.gov.au/covid-19/what-you-can-and-cant-do-under-rules>  <https://web.archive.org/web/20200524123019/https://www.nsw.gov.au/covid-19/travel-and-transport-advice> |
| Health NSW | <https://www.health.nsw.gov.au/Infectious/covid-19/Pages/public-health-orders.aspx> |
|  | <https://www.aljazeera.com/news/2020/6/3/coronavirus-travel-restrictions-border-shutdowns-by-country> |
| Home affairs.gov | <https://web.archive.org/web/20200330015246/https://covid19.homeaffairs.gov.au/travel-restrictions> |
| Education NSW | <https://education.nsw.gov.au/news/latest-news/nsw-students-to-transition-back-to-the-classroom-in-term-2> |
| Healthdirect | <https://www.healthdirect.gov.au/covid19-restriction-checker> |
| COVID-19 Law | <https://thecovid19.law.blog/nsw/nsw-legislation/> |
| Queensland | |
| Health Queensland | <https://www.health.qld.gov.au/system-governance/legislation/cho-public-health-directions-under-expanded-public-health-act-powers/revoked/non-essential-business-closure-direction-23-03-2020>  <https://www.health.qld.gov.au/system-governance/legislation/cho-public-health-directions-under-expanded-public-health-act-powers/revoked/non-essential-business,-activity-and-undertaking-closure-direction-no.2>  <https://www.health.qld.gov.au/system-governance/legislation/cho-public-health-directions-under-expanded-public-health-act-powers/revoked/non-essential-business,-activity-and-undertaking-closure-direction-no.3>  <https://www.health.qld.gov.au/system-governance/legislation/cho-public-health-directions-under-expanded-public-health-act-powers/revoked/non-essential-business-closure-direction-4>  <https://www.health.qld.gov.au/system-governance/legislation/cho-public-health-directions-under-expanded-public-health-act-powers/revoked/non-essential-business-closure-direction-5> <https://www.health.qld.gov.au/system-governance/legislation/cho-public-health-directions-under-expanded-public-health-act-powers/revoked/non-essential-business-closure-direction-6>  <https://www.health.qld.gov.au/system-governance/legislation/cho-public-health-directions-under-expanded-public-health-act-powers/revoked/non-essential-business-closure-direction-7>  <https://www.health.qld.gov.au/system-governance/legislation/cho-public-health-directions-under-expanded-public-health-act-powers/revoked/non-essential-business-closure-direction-8>  <https://www.health.qld.gov.au/news-events/doh-media-releases/releases/queensland-novel-coronavirus-covid-19-update41>  <https://www.health.qld.gov.au/system-governance/legislation/cho-public-health-directions-under-expanded-public-health-act-powers/revoked/movement-and-gathering-direction>  <https://www.health.qld.gov.au/system-governance/legislation/cho-public-health-directions-under-expanded-public-health-act-powers/revoked/movement-gathering-direction-2>  <https://www.health.qld.gov.au/system-governance/legislation/cho-public-health-directions-under-expanded-public-health-act-powers/revoked/movement-gathering-direction-3>  <https://www.health.qld.gov.au/system-governance/legislation/cho-public-health-directions-under-expanded-public-health-act-powers/movement-gathering-direction-4>  <https://www.health.qld.gov.au/system-governance/legislation/cho-public-health-directions-under-expanded-public-health-act-powers/revoked/business-activity-undertaking-direction-6>  <https://www.health.qld.gov.au/system-governance/legislation/cho-public-health-directions-under-expanded-public-health-act-powers/revoked/home-confinement-movement-gathering-direction-1>  <https://www.health.qld.gov.au/system-governance/legislation/cho-phttps://www.health.qld.gov.au/system-governance/legislation/cho-public-health-directions-under-expanded-public-health-act-powers/revoked/border-restrictions-direction-14>  Public-health-directions-under-expanded-public-health-act-powers/revoked/border-restrictions-3  <https://www.health.qld.gov.au/system-governance/legislation/cho-public-health-directions-under-expanded-public-health-act-powers/revoked/home-confinement-movement-gathering-direction-1>  <https://www.health.qld.gov.au/system-governance/legislation/cho-public-health-directions-under-expanded-public-health-act-powers/revoked/movement-and-gathering-direction>  <https://www.health.qld.gov.au/system-governance/legislation/cho-public-health-directions-under-expanded-public-health-act-powers/revoked/border-restrictions-2> |
| COVID-19 Queensland | <https://www.covid19.qld.gov.au/government-actions/roadmap-to-easing-queenslands-restrictions> |
| Queensland Government | <https://web.archive.org/web/20200820022913/https://www.qld.gov.au/health/conditions/health-alerts/coronavirus-covid-19/current-status/public-health-directions/border-restrictions> |
| Queensland teachers union | <https://www.qtu.asn.au/nflash-1120> |
| University of Queensland | <https://about.uq.edu.au/covid-19-student-communication>  <https://about.uq.edu.au/files/3631/COVID%20road%20map.pdf> |
| ABC | <https://www.abc.net.au/news/2020-05-15/coronavirus-queensland-schools-reopen-date-may-25/12248342> |
|  | <https://www.aljazeera.com/news/2020/6/3/coronavirus-travel-restrictions-border-shutdowns-by-country> |
| Healthdirect | <https://www.healthdirect.gov.au/covid19-restriction-checker/activity> |
| Covid-19 Law | <https://thecovid19.law.blog/qld/qld-legislation/> |
| Tasmania | |
| Tasmanian Government Gazette | <http://www.gazette.tas.gov.au/> (PUBLIC HEALTH ACT 1997 DIRECTION UNDER SECTION 16) |
| Coronavirus Tasmania | <https://web.archive.org/web/20200615181508/https://www.coronavirus.tas.gov.au/families-community/gatherings>  <https://www.coronavirus.tas.gov.au/families-community/current-restrictions>  <https://coronavirus.tas.gov.au/__data/assets/pdf_file/0029/96761/17-July-20-Directions-in-Relation-to-Persons-Arriving-in-Tasmania-General.pdf>  <https://www.coronavirus.tas.gov.au/media-releases/our-plan-for-tasmanian-students-to-return-to-the-classroom> |
| Premier Tas | <http://www.premier.tas.gov.au/__data/assets/pdf_file/0020/535115/Our_Plan_to_Rebuild_a_Stronger_Tasmania.pdf>  <http://www.premier.tas.gov.au/releases/nation_leading_border_restrictions_to_protect_tasmanians>  <http://www.premier.tas.gov.au/releases/tasmanias_roadmap_to_recovery>  <http://www.premier.tas.gov.au/releases/additional_coronavirus_management_measures> |
| Tasmania Education Department | <https://www.education.tas.gov.au/2020/05/tasmanian-students-to-return-to-learning-at-school/>  <https://www.education.tas.gov.au/2020/04/school-term-2-update/> |
| Stategrowth Tasmania | <https://www.stategrowth.tas.gov.au/coronavirus> |
| University of Tasmania | <https://www.utas.edu.au/__data/assets/pdf_file/0017/1312253/COVID-update-16-March.pdf>  <https://www.utas.edu.au/__data/assets/pdf_file/0020/1331345/UTAS-RESPONSE-MEASURES-FOR-THE-THREE-STAGES-OF-RETURN-TO-CAMPUS.pdf> |
|  | <https://www.aljazeera.com/news/2020/6/3/coronavirus-travel-restrictions-border-shutdowns-by-country> |
| Healthdirect | <https://web.archive.org/web/20200909171512/https://www.healthdirect.gov.au/covid19-restriction-checker/gatherings-and-work/tas> |
| ABC | <https://www.abc.net.au/news/2020-03-19/coronavirus-tasmanian-premier-announces-border-restrictions/12069764> |
|  | <https://web.archive.org/web/20200330015246/https://covid19.homeaffairs.gov.au/travel-restrictions> |
| COVID-19 Law | <https://thecovid19.law.blog/tas/> |
| South Australia | |
| SA legislation | Direction of the State Co-Ordinator: Non-essential; Business (And Other Gatherings) Closure Direction  <https://legislation.sa.gov.au/web/information/CV19/EMA-CEASED/Emergency%20Management%20(Public%20Activities%20No%202)%20(COVID-19)%20Direction%202020_19.6.2020_CEASED.pdf>  Mass Gatherings Direction (2)  <https://legislation.sa.gov.au/web/information/CV19/PHA-CEASED/Directions%20of%20Chief%20Executive%20of%20the%20Department%20for%20Health%20and%20Wellbeing%20Mass%20Gatherings%20(No%202)_22.3.2020.pdf>  <https://legislation.sa.gov.au/web/information/CV19/EMA-CEASED/Emergency%20Management%20(Gatherings)(COVID-19)%20Direction%202020_28.3.2020_CEASED.pdf>  Non essential business and other gatherings (COVID-19 direction) 2020  Cross Border Travel Direction 2020:  <https://legislation.sa.gov.au/web/information/CV19/EMA-CEASED/Cross-border%20Travel%20Direction_24.3.2020_CEASED.pdf> |
| ABC | <https://www.abc.net.au/news/2020-05-08/sa-to-reopen-as-no-new-coronavirus-cases-recorded/12228898>  <https://www.abc.net.au/news/2020-05-30/coronavirus-restrictions-what-you-can-do-in-sa-from-june-1/12290232> |
| COVID-19 South Australia | <https://www.covid-19.sa.gov.au/latest-news/covid-restrictions-to-ease-from-monday>.  <https://www.covid-19.sa.gov.au/recovery>  <https://www.covid-19.sa.gov.au/latest-news/195879> (roadmap expired)  <https://www.covid-19.sa.gov.au/home>  <https://www.covid-19.sa.gov.au/__data/assets/pdf_file/0003/170724/Emergency-Management-Gatherings-no3-COVID-19-Direction-2020_FINAL.pdf>  [https://web.archive.org/web/20200619115048/https://www.covid-19.sa.gov.au/recovery - tabs__target-217210-2](https://web.archive.org/web/20200619115048/https:/www.covid-19.sa.gov.au/recovery#tabs__target-217210-2)  <https://web.archive.org/web/20200618022520/https://www.covid-19.sa.gov.au/recovery#tabs__target-217210-1>  <https://web.archive.org/web/20200615093500/https://www.covid-19.sa.gov.au/__data/assets/pdf_file/0019/170821/FAQ-Emergency-Management-Gatherings-No-3-COVID-19-Direction-2020.pdf>  <https://web.archive.org/web/20200724032906/https://www.healthdirect.gov.au/covid19-restriction-checker/gatherings-and-work/sa>  <https://www.covid-19.sa.gov.au/__data/assets/pdf_file/0003/170724/Emergency-Management-Gatherings-no3-COVID-19-Direction-2020_FINAL.pdf> |
|  | <https://www.aljazeera.com/news/2020/6/3/coronavirus-travel-restrictions-border-shutdowns-by-country> |
|  | <https://web.archive.org/web/20200330015246/https://covid19.homeaffairs.gov.au/travel-restrictions> |
| Healthdirect | <https://web.archive.org/web/20200724032906/https://www.healthdirect.gov.au/covid19-restriction-checker/gatherings-and-work/sa> |
|  | <https://playandgo.com.au/sa-coronavirus-restrictions-to-ease-from-11-may-2020/> |
| UNISA | <https://www.unisa.edu.au/siteassets/media-centre/docs/march-27-1157.pdf> |
| SA GOV | [https://www.covid-19.sa.gov.au/latest-news/](https://www.covid-19.sa.gov.au/latest-news/pupil-free-days-announced-for-sa-schools) |
| SA Premier | <https://www.premier.sa.gov.au/news/media-releases/news/schools-open-for-term-2> |
| COVID-19 Law | <https://thecovid19.law.blog/sa/sa-legislation/> |
| Northern Territory | |
| Media Release NT Government | <https://newsroom.nt.gov.au/mediaRelease/32113> |
| Coronavirus NT | <https://coronavirus.nt.gov.au/roadmap-new-normal#/stage_2_friday_15_may>  <https://coronavirus.nt.gov.au/roadmap-new-normal#/stage_3_friday_5_june_current>  <https://coronavirus.nt.gov.au/__data/assets/pdf_file/0007/809953/cho-direction-14-gatherings.pdf>  <https://coronavirus.nt.gov.au/__data/assets/pdf_file/0005/806837/CHO-Directions-No-1-SIGNED.PDF>  [coronavirus.nt.gov.au](http://coronavirus.nt.gov.au/)  <https://coronavirus.nt.gov.au/__data/assets/pdf_file/0008/897443/cho-directions-no-39-directions-major-public-events.pdf>  <https://coronavirus.nt.gov.au/roadmap-new-normal#/stage_3_friday_5_june_current>  <https://coronavirus.nt.gov.au/travel/quarantine/hotspots-covid-19> |
| COVID-19 Law | <https://thecovid19.law.blog/act/act-legislation/>  <https://thecovid19.law.blog/nt/nt-legislation/> |
| Education NT | <https://education.nt.gov.au/> |
| Western Australia | |
| WA Emergency operations | <https://ww2.health.wa.gov.au/Articles/A_E/Coronavirus/COVID19-Public-Health-Emergency-Operations-Centre-Bulletins> |
| Legislation | <https://www.wa.gov.au/government/document-collections/covid-19-coronavirus-revoked-or-superseded-directions> |
| WA gov | <https://www.wa.gov.au/government/document-collections/covid-19-coronavirus-revoked-or-superseded-directions> |
| Australian Capital Territory | |
| COVID-19 ACT | <https://www.covid19.act.gov.au/__data/assets/pdf_file/0008/1561184/CV_Roadmap_Recovery-plan_ease-of-restrictions_26052020.pdf>  <https://www.covid19.act.gov.au/__data/assets/pdf_file/0006/1629330/PICC0050-COVID-Recovery-Plan.pdf> |
| Legislation ACT | <https://www.legislation.act.gov.au/View/ni/2020-169/20200323-73519/PDF/2020-169.PDF>  <https://www.legislation.act.gov.au/View/ni/2020-255/20200501-73888/PDF/2020-255.PDF>  <https://www.legislation.act.gov.au/View/ni/2020-533/20200901-75020/PDF/2020-533.PDF>  <https://www.legislation.act.gov.au/View/ni/2020-617/current/PDF/2020-617.PDF>  <https://www.legislation.act.gov.au/View/ni/2020-387/20200703-74556/PDF/2020-387.PDF>  <https://www.legislation.act.gov.au/View/ni/2020-268/20200508-73930/PDF/2020-268.PDF>  <https://www.legislation.act.gov.au/View/ni/2020-313/20200529-74159/PDF/2020-313.PDF>  <https://www.legislation.act.gov.au/View/ni/2020-533/20200901-75020/PDF/2020-533.PDF>  <https://www.legislation.act.gov.au/ni/2020-177/> |
| Healthdirect | <https://web.archive.org/web/20200625025249/https://www.healthdirect.gov.au/covid19-restriction-checker>  https://web.archive.org/web/20200708055542/https://www.healthdirect.gov.au/covid19-restriction-checker/gatherings-and-work/act /gatherings-and-work/act |
|  | [https://www.aljazeera.com/news/2020/6/3/coronavirus-travel-restrictions-border-shutdowns-by-country -74556/PDF/2020-387.PDF](https://www.aljazeera.com/news/2020/6/3/coronavirus-travel-restrictions-border-shutdowns-by-country%20-74556/PDF/2020-387.PDF) |
|  | <https://web.archive.org/web/20200330015246/https://covid19.homeaffairs.gov.au/travel-restrictions> |
| ANU | <https://www.anu.edu.au/news/all-news/covid-19-update-anu-campuses-move-to-remote-work-and-study>  <https://www.anu.edu.au/news/all-news/covid-19-update-anu-campuses-move-to-remote-work-and-study> |
| Canberra Weekly | <https://canberraweekly.com.au/canberra-universities-heading-back-on-campus/> |
| Education ACT | <https://www.education.act.gov.au/public-school-life/covid-school-arrangements/frequently-asked-questions-for-school-communities>  <https://www.education.act.gov.au/about-us/all-news-and-news-alerts/news-items/may-2020/back-to-the-classroom-joy,-relief-and-even-a-ukelele>  <https://www.education.act.gov.au/about-us/all-news-and-news-alerts/news-items/april-2020/term-2-in-act-public-schools>  <https://www.education.act.gov.au/about-us/all-news-and-news-alerts/news-items/march-2020/act-public-schools-pupil-free-from-tuesday-24-march-to-school-holidays> |

**Table S5**

*Sources used for Objective Social Restriction Recording across the United Kingdom*

| England | |
| --- | --- |
| Source | URL |
| UK Government announcement | <https://www.gov.uk/government/news/government-announces-further-measures-on-social-distancing> |
| UK Government publications | <https://www.gov.uk/government/publications/our-plan-to-rebuild-the-uk-governments-covid-19-recovery-strategy/our-plan-to-rebuild-the-uk-governments-covid-19-recovery-strategy#our-roadmap-to-lift-restrictions-step-by-step>  <https://www.gov.uk/government/publications/actions-for-schools-during-the-coronavirus-outbreak/guidance-for-full-opening-schools>  <https://web.archive.org/web/20200817035044/https://www.gov.uk/government/publications/coronavirus-outbreak-faqs-what-you-can-and-cant-do/coronavirus-outbreak-faqs-what-you-can-and-cant-do> |
| UK Legislation | <https://www.legislation.gov.uk> |
| Institute for Government | <https://www.instituteforgovernment.org.uk/explainers/coronavirus-lockdown-rules-four-nations-uk> |
| BBC | <https://www.bbc.co.uk/news/uk-52984687>  <https://www.bbc.com/news/uk-52774854>  <https://www.bbc.com/news/uk-53358870>  <https://www.bbc.com/news/explainers-53221896>  <https://www.bbc.com/news/uk-51952314> |
| UK Health Act | "The Health Protection (Coronavirus, Wearing of Face Coverings in a Relevant Place) (England) Regulations 2020 |
| UK Gov speech archive | <https://www.gov.uk/government/speeches/pm-statement-on-coronavirus-16-march-2020>  health-directions-under-expanded-public-health-aked/border-restrictions-3 |
| Metro.co.uk | <https://metro.co.uk/2020/03/23/gatherings-two-banned-uk-enters-coronavirus-lockdown-12445478/> |
| UK police | <https://web.archive.org/web/20200617165721/https://www.met.police.uk/advice/advice-and-information/c19/coronavirus-covid-19/coronavirus-social-distancing-rules-england/>  <https://web.archive.org/web/20200704015526/https://www.met.police.uk/advice/advice-and-information/c19/coronavirus-covid-19/coronavirus-social-distancing-rules-england/>  [www.met.police.uk/advice/advice-and-information/c19/coronavirus-covid-19/coronavirus-social-distancing-rules-england/](http://www.met.police.uk/advice/advice-and-information/c19/coronavirus-covid-19/coronavirus-social-distancing-rules-england/) |
| UK GOV Archive | [https://web.archive.org/web/20200613131448/https://www.gov.uk/guidance/meeting-people-from-outside-your-household](https://web.archive.org/web/20200613131448/https:/www.gov.uk/guidance/meeting-people-from-outside-your-household)  <https://www.gov.uk/guidance/coronavirus-covid-19-countries-and-territories-exempt-from-advice-against-all-but-essential-international-travel>  <https://web.archive.org/web/20200320132147/https://www.gov.uk/guidance/coronavirus-covid-19-information-for-the-public> |
| UK Government news | <https://www.gov.uk/government/news/travel-advice-foreign-secreatary-statement-17-march-2020>  <https://www.gov.uk/government/news/pupils-start-returning-to-schools-in-england> |
| UK Guardian | <https://www.theguardian.com/world/2020/mar/23/uk-lockdown-what-are-new-coronavirus-restrictions> |
| NHS | <https://web.archive.org/web/20200423115737/https://www.nhs.uk/conditions/coronavirus-covid-19/what-to-do-if-you-or-someone-you-live-with-has-coronavirus-symptoms/staying-at-home-if-you-or-someone-you-live-with-has-coronavirus-symptoms/>  <https://www.nhs.uk/conditions/coronavirus-covid-19/>  <https://web.archive.org/web/20200528203403/https://www.gov.uk/guidance/nhs-test-and-trace-how-it-works> |
| Northern Ireland | |
| NI Gov Health archive | <https://web.archive.org/web/20200418052002/https://www.health-ni.gov.uk/sites/default/files/publications/health/Coronavirus-Restrictiions-Regs-2020.pdf>  <https://web.archive.org/web/20200418052002/https://www.health-ni.gov.uk/sites/default/files/publications/health/Coronavirus-Restrictiions-Regs-2020.pdf>  <https://web.archive.org/web/20200813090943/https://www.nidirect.gov.uk/articles/coronavirus-covid-19-advice-schools-colleges-and-universities> |
| NI Legislation | <https://www.legislation.gov.uk/nisr/2020/55/regulation/3/2020-03-28>  <https://www.legislation.gov.uk/nisr/2020/55/2020-05-19>  <https://www.legislation.gov.uk/nisr/2020/55/2020-06-11>  <https://www.legislation.gov.uk/nisr/2020/55/2020-06-29> |
| BBC | <https://www.bbc.com/news/uk-northern-ireland-52925516>  <https://www.bbc.com/news/uk-northern-ireland-52978413>  <https://www.bbc.com/news/uk-northern-ireland-53007269>  <https://www.bbc.com/news/uk-53358870>  <https://www.bbc.com/news/explainers-53221896>  <https://www.bbc.com/news/uk-northern-ireland-51923280> |
| NI Direct | <https://www.nidirect.gov.uk/> |
| UK Gov Travel Advice | <https://www.gov.uk/government/news/travel-advice-foreign-secreatary-statement-17-march-2020> |
| Wales | |
| GOV Wales | <https://gov.wales/coronavirus-regulations-guidance>  <https://web.archive.org/web/20200329005529/https://gov.wales/coronavirus-regulations-guidance>  <https://gov.wales/wales-extends-coronavirus-lockdown>  <https://web.archive.org/web/20200620163139/https://gov.wales/coronavirus-regulations-changes-monday-22-june>  <https://web.archive.org/web/20200714041554/https://gov.wales/coronavirus-regulations-guidance>  <https://web.archive.org/web/20200803154857/https://gov.wales/coronavirus-regulations-guidance#section-39241>  <https://web.archive.org/web/20200824152600/https://gov.wales/coronavirus-regulations-guidance#section-39237>  <https://web.archive.org/web/20200917040323/https://gov.wales/coronavirus-regulations-guidance>  <https://web.archive.org/web/20200925161547/https://gov.wales/coronavirus-regulations-guidance>  <https://web.archive.org/web/20200518095436/https://gov.wales/coronavirus-regulations-guidance#section-39239>  <https://web.archive.org/web/20200610150847/https://gov.wales/how-self-isolate-when-you-travel-wales-coronavirus-covid-19> |
| BBC | <https://www.bbc.co.uk/news/live/uk-wales-51994269>  <https://www.bbc.co.uk/news/uk-wales-52584690>  <https://www.bbc.co.uk/news/uk-wales-52584690>  <https://www.bbc.co.uk/news/uk-wales-52842327>  <https://www.bbc.com/news/uk-52774854>  <https://www.bbc.com/news/uk->  <https://www.bbc.com/news/uk-51928400>  https://www.bbc.com/news/uk-wales-54167804  <https://www.bbc.com/news/uk-wales-54167804> |
| Gov Wales Statement | <https://gov.wales/statement-minster-education-kirsty-williams-school-closures-wales> |
| Scotland | |
| Gov Scot News | <https://www.gov.scot/news/pubs-cafes-and-more-told-to-close/>  <https://www.gov.scot/news/effective-lockdown-to-be-introduced/>  https://www.gov.scot/news/ |
| Gov Scot publications | https://www.gov.scot/publications/  <https://www.gov.scot/publications/first-minister-covid-19-update-1/>  <https://www.gov.scot/publications/coronavirus-covid-19-update-first-ministers-speech-6-july-2020/>  <https://www.gov.scot/publications/coronavirus-covid-19-phase-3-staying-safe-and-protecting-others/pages/shopping-eating-and-drinking-out/>  <https://web.archive.org/web/20200809032955/https://www.gov.scot/publications/coronavirus-covid-19-what-you-can-and-cannot-do/pages/shopping-eating-and-drinking-out/>  <https://web.archive.org/web/20200606171702if_/https://www.gov.scot/publications/coronavirus-covid-19-what-you-can-and-cannot-do/pages/seeing-friends-and-family/>  <https://web.archive.org/web/20200705113524/https://www.gov.scot/publications/coronavirus-covid-19-what-you-can-and-cannot-do/pages/seeing-friends-and-family/>  <https://www.gov.scot/publications/coronavirus-covid-19-universities-colleges-and-student-accommodation-providers/pages/health-and-safety/>  <https://web.archive.org/web/20200809032549/https://www.gov.scot/publications/coronavirus-covid-19-what-you-can-and-cannot-do/pages/seeing-friends-and-family/>  <https://web.archive.org/web/20200926212631/https://www.gov.scot/publications/coronavirus-covid-19-phase-3-staying-safe-and-protecting-others/pages/seeing-friends-and-family/>  <https://web.archive.org/web/20200815141932/https://www.gov.scot/publications/coronavirus-covid-19-what-you-can-and-cannot-do/pages/schools-and-childcare-settings/> |
| Scot government guidance | <https://www.gov.scot/collections/coronavirus-covid-19-guidance/>  <https://www.gov.uk/guidance/coronavirus-covid-19-countries-and-territories-exempt-from-advice-against-all-but-essential-international-travel> |
| BBC | <https://www.bbc.com/news/uk-scotland-53354308>  <https://www.bbc.com/news/uk-scotland-51899757>  https://www.gov.scot/news/effective-lockdown-to-be-introduced/  <https://www.bbc.com/news/uk-scotland-52745643>  <https://www.bbc.com/news/uk-scotland-53340520>  <https://www.bbc.com/news/uk-scotland-52808586>  <https://www.bbc.com/news/uk-52774854>  <https://www.bbc.com/news/uk-53358870>  <https://www.bbc.com/news/explainers-53221896>  <https://www.bbc.com/news/uk-scotland-51880666>  <https://www.bbc.com/news/uk-51928400> |
|  | <https://www.gov.uk/government/news/travel-advice-foreign-secreatary-statement-17-march-2020> |

**Table S6**

*Sources used for Objective Social Restriction Recording across the United States*

| Source | URL |
| --- | --- |
| Oxford Database | <https://www.bsg.ox.ac.uk/research/research-projects/covid-19-government-response-tracker> |
| COVID-19 State Policy^5^ | [https://github.com/COVID19StatePolicy/SocialDistancing/tree/master/source](https://protect-au.mimecast.com/s/BcToCyojKAsroGwHMnm4d?domain=github.com) |
| Education Week | [https://www.edweek.org/leadership/map-where-are-schools-closed/2020/07](https://protect-au.mimecast.com/s/aEZpCzvkL4uMqXZigmJte?domain=edweek.org) |
| Alabama State University | <https://www.alabamanews.net/2020/03/12/asu-closing-campus-for-remainder-of-spring-semester-following-spring-break-vacation/> |
| University of Alaska | <https://sites.google.com/alaska.edu/coronavirus/general-info/status> |
| Henderson State University, Arkansas | <https://hsu.edu/pages/coronavirus/> |
| Arizona State University | <https://eoss.asu.edu/health/announcements/coronavirus> |
| California State University | <http://www.calstatela.edu/healthwatch> |
| Colorado State University | <https://covid.colostate.edu/> |
| Wesleyan University, Connecticut | <https://www.wesleyan.edu/healthservices/coronavirus/> |
| University of the District of Columbia | <http://docs.udc.edu/president/UDC-COVID-19_Memo_Update-3-13-20-final.pdf> |
| Florida State University | <https://www.flbog.edu/2020/03/11/state-university-system-statement-on-covid-19/> |
| Georgia State University | <https://www.usg.edu/coronavirus> |
| University of Hawaii | <https://www.hawaiinewsnow.com/2020/03/12/coronavirus-fears-grow-uh-prepares-possibility-taking-all-classes-online/> |
| Iowa State University | <https://web.iastate.edu/safety/updates/covid19> |
| Boise State University, Idaho | <https://www.boisestate.edu/publicsafety-emergencymanagement/2020/03/10/campus-wide-emergency-preparation-set-for-friday-mar-13/> |
| Illinois State University | <https://news.illinoisstate.edu/2020/03/coronavirus-message-from-president-dietz/> |
| Indiana State University | <https://www.indstate.edu/covid> |
| Kansas State University | <https://www.k-state.edu/covid-19/> |
| Kentucky State University | <https://kysu.edu/2020/03/12/presidential-plain-speak-regarding-ksu-covid-19/> |
| Louisiana State University | <https://lsu.edu/coronavirus/> |
| University of Massachusetts | <https://www.massachusetts.edu/sites/default>  /files/documents/20.3.11%20COVID-19%20BOT%20FINAL.pdf |
| University of Maryland | <https://www.wbaltv.com/article/university-system-of->  maryland-schools-to-prepare-for-remote-instruction-following-spring-break/31351840 |
| University of Maine | <https://umaine.edu/coronavirus/> |
| Michigan State University | <https://msu.edu/coronavirus/> |
| Minnesota State University | <https://www.mnstate.edu/emergency/covid-19/> |
| Mississippi State University | <https://www.wtva.com/content/news/IHL-extends-spring-break>  -at-Mississippi-universities-over-coronavirus-concerns-568740711.html |
| Missouri State University | <https://www.missouristate.edu/Coronavirus/> |
| Montana State University | <https://www.msun.edu/news/article.aspx?id=19552/info-on-the-coronavirus> |
| Nevada State College | <https://nsc.edu> |
| New Mexico State University | https://www.nmsu.edu |
| University of Nebraska | <https://nebraska.edu/news-and-events/news/2020/03/university-of->  nebraska-campuses-to-transition-to-remote-learning-after-spring-break |
| University of New Hampshire | <https://manchester.unh.edu/coronavirus-covid-19-updates#collapse_4134> |
| Montclair State University, New Jersey | <https://www.montclair.edu/university-health-center/coronavirus/message-from-president-cole/> |
| State University of New York | <https://www.farmingdale.edu/health-wellness-center/coronavirus/index.shtml> |
| North Carolina State University | <https://www.ncsu.edu> |
| North Dakota State University | <https://www.ndsu.edu/police_safety/news/detail/57168/> |
| Kent State University, Ohio | <https://www.kent.edu/coronavirus> |
| Oklahoma State University | <https://go.okstate.edu/coronavirus/latest-announcements/> |
| Oregon State University | <https://leadership.oregonstate.edu/coronavirus> |
| Pennsylvania State University | <https://virusinfo.psu.edu/> |
| South Carolina State University | <https://www.scsu.edu/news_article.aspx?news_id=2145> |
| South Dakota State University | <https://www.sdstate.edu/safety-security/emergency-management/coronavirus-updates> |
| Tennessee State University | <http://www.tnstate.edu/campus_life/tornadorelief.aspx> |
| Texas State University | <https://www.universitystar.com/news/breaking/texas-state-joins-surrounding-universities>  -in-taking-action-on-coronavirus-concerns/article_a59e20da-4546-537b-8430-6a0134127d0b.html |
| Utah State University | <https://www.usu.edu/today/story/update-for-usu-community-on-coronavirus> |
| Virgin Islands | <https://doh.vi.gov/sites/default/files/DOE%20-%20REVISED%20->  %20Reopening%20of%20Public%20Schools%20Delayed%  20One%20Week%2C%20Will%20Open%20on%20Sept%2014.pdf |
| Virginia State University | <http://www.vsu.edu/news/news/2020/VSU-Coronavirus-Updates.php> |
| West Virginia State University | <https://www.wvstateu.edu> |
| Norwich University, Vermont | <https://www.norwich.edu/news/psa> |
| University of Wisconsin | <https://uwosh.edu/coronavirus/> |
| University of Wyoming | <http://www.uwyo.edu/uw/news/2020/03/uw-moves-to-online-course-delivery-for-remainder-of-spring-semester.html> |

*Note.* ^5^State government websites were individually accessed through this source

**Table S7**

*Estimated Sample Statistics and Model Results for Model SA1:* ***Sensitivity Analyses 1*** *(LGCM for All of Sample without social restrictions in the model; includes predictors).*

|  | **Estimated Sample Statistics** | | | | | | | | | | | | | |
| --- | --- | --- | --- | --- | --- | --- | --- | --- | --- | --- | --- | --- | --- | --- |
|  | **Means** | **Loneliness** | | | | | **Depression** | | | | **Social Anxiety** | | | |
|  |  | T1 | | T2 | T3 | | T1 | T2 | T3 | | T1 | T2 | | T3 |
|  |  | 45.724 | | 45.978 | 46.076 | | 8.379 | 7.795 | 7.859 | | 3.712 | 3.753 | | 3.916 |
|  | Covariances | Loneliness | | | | | Depression | | | | Social Anxiety | | | |
|  |  | T1 | | T2 | T3 | | T1 | T2 | T3 | | T1 | T2 | | T3 |
| Loneliness | T1 | 124.928 | |  |  | |  |  |  | |  |  | |  |
|  | T2 | 111.143 | | 137.185 |  | |  |  |  | |  |  | |  |
|  | T3 | 112.909 | | 123.700 | 143.956 | |  |  |  | |  |  | |  |
| Depression | T1 | 36.368 | | 36.442 | 37.774 | | 35.344 |  |  | |  |  | |  |
|  | T2 | 33.474 | | 41.071 | 39.670 | | 26.016 | 33.726 |  | |  |  | |  |
|  | T3 | 33.843 | | 38.206 | 43.672 | | 25.408 | 26.791 | 34.845 | |  |  | |  |
| Social Anxiety | T1 | 17.042 | | 16.580 | 16.778 | | 8.957 | 7.550 | 7.560 | | 10.084 |  | |  |
|  | T2 | 14.995 | | 17.479 | 17.586 | | 8.160 | 9.079 | 8.431 | | 7.242 | 9.928 | |  |
|  | T3 | 17.237 | | 19.054 | 20.216 | | 8.638 | 8.761 | 9.534 | | 7.705 | 7.772 | | 10.844 |
|  | **Correlations** | | | | | | | | | | | | | |
|  |  | **Loneliness** | | | | | **Depression** | | | | **Social Anxiety** | | | |
|  |  | T1 | | T2 | T3 | | T1 | T2 | T3 | | T1 | T2 | | T3 |
| Loneliness | T1 | 1.00 | |  |  | |  |  |  | |  |  | |  |
|  | T2 | 0.849 | | 1.00 |  | |  |  |  | |  |  | |  |
|  | T3 | 0.842 | | 0.880 | 1.00 | |  |  |  | |  |  | |  |
| Depression | T1 | 0.547 | | 0.523 | 0.530 | | 1.00 |  |  | |  |  | |  |
|  | T2 | 0.516 | | 0.604 | 0.569 | | 0.754 | 1.00 |  | |  |  | |  |
|  | T3 | 0.513 | | 0.553 | 0.617 | | 0.724 | 0.781 | 1.00 | |  |  | |  |
| Social Anxiety | T1 | 0.480 | | 0.446 | 0.440 | | 0.474 | 0.409 | 0.403 | | 1.00 |  | |  |
|  | T2 | 0.426 | | 0.474 | 0.465 | | 0.436 | 0.496 | 0.453 | | 0.724 | 1.00 | |  |
|  | T3 | 0.468 | | 0.494 | 0.512 | | 0.441 | 0.458 | 0.490 | | 0.737 | 0.749 | | 1.00 |
|  | **Model Results** | | | | | | | | | | | | | |
|  |  | | **Estimate** | | | **Standard Error (*SE*)** | | | | **Estimate/*SE*** | | | ***p*-value** | |
|  | Intercept Loneliness | | 46.035 | | | 0.221 | | | | 208.305 | | | <0.001 | |
|  | Slope Loneliness | | -0.471 | | | 0.033 | | | | -14.327 | | | <0.001 | |
|  | Intercept Depression | | 8.308 | | | 0.115 | | | | 72.450 | | | <0.001 | |
|  | Slope Depression | | -0.086 | | | 0.020 | | | | -4.203 | | | <0.001 | |
|  | Intercept Social Anxiety | | 3.443 | | | 0.057 | | | | 60.442 | | | <0.001 | |
|  | Slope Social Anxiety | | 0.653 | | | 0.014 | | | | 47.560 | | | <0.001 | |

**Table S8**

*Sample Parameter Estimates for Model SA1: Estimated Sample Statistics and Model Results for Model 2b****: Sensitivity Analyses 1*** *(LGCM for All of sample without social restrictions in the model; includes predictors).*

| **Model Results** | | | | |
| --- | --- | --- | --- | --- |
|  | **Estimate** | **Standard Error (*SE*)** | **Estimate/*SE*** | ***p*-value** |
| Intercept of Loneliness ⭢ Slope of Loneliness | -0.22 | 0.07 | -3.05 | 0.002 |
| Intercept of Depression ⭢ |  |  |  |  |
| Slope of Depression | -0.18 | 0.11 | -1.70 | 0.09 |
| Intercept of Loneliness | 0.62 | 0.03 | 21.85 | <0.001 |
| Slope of Loneliness | -0.34 | 0.10 | -3.25 | 0.001 |
| Intercept of Social Anxiety ⭢ |  |  |  |  |
| Slope of Social Anxiety | 1.77 | 0.51 | 3.46 | 0.001 |
| Intercept of Loneliness | 0.60 | 0.03 | 23.15 | <0.001 |
| Slope of Loneliness | -0.45 | 0.12 | -3.80 | <0.001 |
| Intercept for Depression | 0.62 | 0.03 | 21.10 | <0.001 |
| Slope of Depression | -0.10 | 0.09 | -1.10 | 0.27 |
| Slope of Depression ⭢ |  |  |  |  |
| Intercept of Loneliness | -0.10 | 0.08 | -1.25 | 0.21 |
| Slope of Loneliness | 1.09 | 0.44 | 2.50 | 0.01 |
| Slope of Social Anxiety ⭢ |  |  |  |  |
| Intercept of Loneliness | 0.97 | 0.26 | 3.74 | <0.001 |
| Slope of Loneliness | -0.58 | 0.25 | -2.30 | 0.02 |
| Intercept of Depression | 0.90 | 0.24 | 3.77 | <0.001 |
| Slope of Depression | 0.29 | 0.18 | 1.55 | 0.12 |

**Table S9**

*Estimated Sample Statistics and Model Results for Model SA2:* ***Sensitivity Analyses 2*** *(LGCM for sub-sample where only data from participants with complete data T1-T3; model includes predictor variables).*

|  | **Estimated Sample Statistics** | | | | | | | | | | | | | |
| --- | --- | --- | --- | --- | --- | --- | --- | --- | --- | --- | --- | --- | --- | --- |
|  | **Means** | **Loneliness** | | | | **Depression** | | | **Social Anxiety** | | | **Restrictions Severity** | | |
|  |  | T1 | | T2 | T3 | T1 | T2 | T3 | T1 | T2 | T3 | T1 | T2 | T3 |
|  |  | 44.418 | | 44.712 | 44.753 | 7.472 | 7.072 | 7.309 | 3.435 | 3.442 | 3.622 | 22.976 | 19.617 | 18.575 |
|  | **Covariances** | **Loneliness** | | | | **Depression** | | | **Social Anxiety** | | | **Restrictions Severity** | | |
|  |  | T1 | | T2 | T3 | T1 | T2 | T3 | T1 | T2 | T3 | T1 | T2 | T3 |
| Loneliness | T1 | 128.046 | |  |  |  |  |  |  |  |  |  |  |  |
|  | T2 | 114.572 | | 137.730 |  |  |  |  |  |  |  |  |  |  |
|  | T3 | 115.693 | | 124.667 | 146.687 |  |  |  |  |  |  |  |  |  |
| Depression | T1 | 35.644 | | 35.104 | 36.281 | 30.610 |  |  |  |  |  |  |  |  |
|  | T2 | 34.860 | | 41.187 | 38.878 | 24.084 | 31.517 |  |  |  |  |  |  |  |
|  | T3 | 34.782 | | 37.803 | 43.617 | 23.230 | 25.082 | 33.209 |  |  |  |  |  |  |
| Social Anxiety | T1 | 16.338 | | 16.095 | 15.987 | 8.046 | 7.377 | 7.678 | 9.479 |  |  |  |  |  |
|  | T2 | 15.342 | | 17.586 | 17.533 | 7.548 | 8.598 | 8.303 | 6.825 | 9.271 |  |  |  |  |
|  | T3 | 17.145 | | 18.369 | 19.319 | 8.158 | 8.028 | 9.384 | 7.420 | 7.447 | 10.387 |  |  |  |
| Restrictions Severity | T1 | -2.246 | | -2.624 | -1.585 | 0.412 | 0.762 | 0.013 | -0.270 | -0.201 | -0.619 | 15.277 |  |  |
|  | T2 | 0.682 | | -0.856 | -1.554 | -.0303 | 0.592 | -0.334 | 0.009 | -0.180 | -0.321 | 5.304 | 6.030 |  |
|  | T3 | -2.943 | | -3.270 | -2.762 | 0.156 | 1.229 | 0.759 | 0.286 | 0.487 | 0.262 | 13.659 | 8.105 | 29.586 |
| **Correlations** | | | | | | | | | | | | | | |
|  |  | **Loneliness** | | | | **Depression** | | | **Social Anxiety** | | | **Restrictions Severity** | | |
|  |  | T1 | | T2 | T3 | T1 | T2 | T3 | T1 | T2 | T3 | T1 | T2 | T3 |
| Loneliness | T1 | 1.00 | |  |  |  |  |  |  |  |  |  |  |  |
|  | T2 | 0.863 | | 1.00 |  |  |  |  |  |  |  |  |  |  |
|  | T3 | 0.844 | | 0.877 | 1.00 |  |  |  |  |  |  |  |  |  |
| Depression | T1 | 0.569 | | 0.541 | 0.541 | 1.00 |  |  |  |  |  |  |  |  |
|  | T2 | 0.549 | | 0.625 | 0.572 | 0.775 | 1.00 |  |  |  |  |  |  |  |
|  | T3 | 0.533 | | 0.559 | 0.625 | 0.729 | 0.775 | 1.00 |  |  |  |  |  |  |
| Social Anxiety | T1 | 0.469 | | 0.445 | 0.429 | 0.472 | 0.427 | 0.433 | 1.00 |  |  |  |  |  |
|  | T2 | 0.445 | | 0.492 | 0.475 | 0.448 | 0.503 | 0.473 | 0.728 | 1.00 |  |  |  |  |
|  | T3 | 0.470 | | 0.486 | 0.495 | 0.458 | 0.444 | 0.505 | 0.748 | 0.759 | 1.00 |  |  |  |
| Restriction Severity | T1 | -0.051 | | -0.057 | -0.033 | 0.019 | 0.035 | 0.001 | -0.022 | -0.017 | -0.049 | 1.00 |  |  |
|  | T2 | -0.025 | | -0.030 | -0.052 | -0.022 | 0.043 | -0.024 | 0.001 | -0.024 | -0.041 | 0.553 | 1.00 |  |
|  | T3 | -0.048 | | -0.051 | -0.042 | -0.005 | 0.040 | 0.024 | 0.017 | 0.029 | 0.015 | 0.642 | 0.607 | 1.00 |
|  | **Model Results** | | | | | | | | | | | | | |
|  |  | | **Estimate** | | | **Standard Error (*SE*)** | | | **Estimate/*SE*** | | | ***p*-value** | | |
|  | Intercept Loneliness | | 44.986 | | | 0.443 | | | 101.553 | | | <0.001 | | |
|  | Slope Loneliness | | -.0456 | | | 0.045 | | | -10.088 | | | <0.001 | | |
|  | Intercept Depression | | 7.368 | | | 0.207 | | | 35.644 | | | <0.001 | | |
|  | Slope Depression | | -0.027 | | | 0.026 | | | -1.053 | | | 0.292 | | |
|  | Intercept Social Anxiety | | 3.010 | | | 0.098 | | | 30.756 | | | <0.001 | | |
|  | Slope Social Anxiety | | 0.584 | | | 0.020 | | | 29.041 | | | <0.001 | | |
|  | Intercept Social Restrictions | | 21.979 | | | 0.097 | | | 226.127 | | | <0.001 | | |
|  | Slope Social Restrictions | | -0.813 | | | 0.025 | | | -32.659 | | | <0.001 | | |

*Note.* Model fit statistics = RMSEA = 0.077 [0.070, 0.083], CFI = 0.935, TLI = 0.879, SRMR = 0.046

**Table S10**

*Sample Parameter Estimates for Model SA2:* ***Sensitivity Analyses 2*** *(LGCM for sub-sample where only data from participants with complete data T1-T3; model includes predictor variables).*

| **Model Results** | | | | |
| --- | --- | --- | --- | --- |
|  | **Estimate** | **Standard Error (*SE*)** | **Estimate/*SE*** | ***p*-value** |
| Intercept of Loneliness ⭢ Slope of Loneliness | -0.15 | 0.11 | -1.44 | 0.149 |
| Intercept of Depression ⭢ |  |  |  |  |
| Slope of Depression | -0.10 | 0.14 | -0.74 | 0.457 |
| Intercept of Loneliness | 0.67 | 0.04 | 18.48 | <0.0001 |
| Slope of Loneliness | -0.39 | 0.21 | -1.88 | 0.06 |
| Intercept of Social Anxiety ⭢ |  |  |  |  |
| Slope of Social Anxiety | 2.12 | 0.85 | 2.49 | 0.01 |
| Intercept of Loneliness | 0.60 | 0.04 | 16.95 | <0.001 |
| Slope of Loneliness | -0.60 | 0.296 | -2.04 | 0.04 |
| Intercept for Depression | 0.58 | 0.04 | 14.78 | <0.001 |
| Slope of Depression | 0.07 | 0.09 | 0.81 | 0.42 |
| Slope of Depression ⭢ |  |  |  |  |
| Intercept of Loneliness | -.04 | 0.09 | -.46 | 0.64 |
| Slope of Loneliness | 1.28 | 0.79 | 1.63 | 0.104 |
| Slope of Social Anxiety ⭢ |  |  |  |  |
| Intercept of Loneliness | 1.124 | 0.42 | 2.67 | 0.008 |
| Slope of Loneliness | -0.98 | 0.64 | -1.53 | 0.13 |
| Intercept of Depression | 1.02 | 0.38 | 2.66 | 0.008 |
| Slope of Depression | 0.52 | 0.32 | 1.61 | 0.11 |
| Intercept of Social Restrictions ⭢ |  |  |  |  |
| Slope of Social Restrictions | 3.26 | 0.19 | 17.59 | <0.001 |
| Intercept of Loneliness | -0.11 | 0.14 | -0.78 | 0.44 |
| Slope of Loneliness | -0.02 | 0.02 | -1.37 | 0.17 |
| Intercept of Depression | -0.09 | 0.07 | -1.18 | 0.24 |
| Slope of Depression | 0.002 | 0.01 | 0.16 | 0.88 |
| Intercept of Social Anxiety | -0.02 | 0.04 | -0.43 | 0.67 |
| Slope of Social Anxiety | -0.008 | 0.008 | -1.07 | 0.29 |
| Slope of Social Restrictions ⭢ |  |  |  |  |
| Intercept of Loneliness | -0.39 | 0.47 | -0.85 | 0.40 |
| Slope of Loneliness | -0.04 | 0.05 | -0.76 | 0.45 |
| Intercept of Depression | -0.14 | 0.25 | -0.58 | 0.56 |
| Slope of Depression | -0.02 | 0.03 | -0.71 | 0.48 |
| Intercept of Social Anxiety | -0.12 | 0.11 | -1.04 | 0.30 |
| Slope of Social Anxiety | -0.05 | 0.02 | -2.09 | 0.04 |

**Table S11**

*Demographics for subsample across Australia, United Kingdom, and United States*

| Item |  | Australia | United Kingdom | United States | Overall |
| --- | --- | --- | --- | --- | --- |
| *N* | | 701 | 483 | 378 | 1562 |
| Gender *n* (%) | |  |  |  |  |
|  | Male | 120 (17.1%) | 54 (12.2%) | 43 (11.4%) | 217 (13.9%) |
|  | Female | 566 (80.9%) | 424 (87.8%) | 325 (86.0%) | 1315 (84.2%) |
| Age | |  |  |  |  |
|  | Mean age | 44.20 | 52.19 | 52.94 | 48.80 |
|  | Range | 18-81 | 19-91 | 18-88 | 18-91 |
| Relationship status *n* (%) | |  |  |  |  |
|  | In a relationship/married | 438 (62.7%) | 305 (63.1%) | 231 (61.1%) | 974 (62.4%) |
|  | Single (including separated/divorced/widowed) | 251 (35.9%) | 173 (35.8%) | 143 (37.8%) | 567 (36.3%) |
|  | Other | 10 (1.4%) | 5 (1%) | 4 (1.1%) | 19 (1.1%) |
| Work status *n* (%) | |  |  |  |  |
|  | Full-time | 347 (49.6%) | 171 (35.4%) | 166 (43.9%) | 684 (43.8%) |
|  | Part-time/casual/self-employed | 205 (29.3%) | 113 (23.4%) | 61 (16.1%) | 379 (24.3%) |
|  | Student | 49 (7.0%) | 18 (3.7%) | 17 (4.5%) | 84 (5.4%) |
|  | Unemployed | 38 (5.4%) | 49 (10.1%) | 34 (9.0%) | 121 (7.7%) |
|  | Retired | 42 (6.0%) | 110 (22.8%) | 91 (24.1%) | 243 (15.6%) |
|  | Other | 19 (2.7%) | 22 (4.6%) | 9 (2.4%) | 50 (3.2%) |
| Household status *n* (%) | |  |  |  |  |
|  | Living with family | 505 (72.1%) | 342 (70.8%) | 260 (69.0%) | 1107 (70.9%) |
|  | Living alone | 144 (20.6%) | 116 (24.0%) | 95 (25.2%) | 355 (22.7%) |
|  | Other, i.e., living with non-family members | 51 (7.3%) | 25 (5.2%) | 22 (5.8%) | 98 (6.3%) |
| Financial status *n* (%) | |  |  |  |  |
|  | Very well | 312 (44.7%) | 189 (39.1%) | 138 (36.5%) | 639 (41%) |
|  | Fairly well | 331 (47.4%) | 248 (51.3%) | 181 (47.9%) | 760 (48.7%) |
|  | Poorly | 55 (7.8%) | 46 (9.5%) | 59 (15.6%) | 160 (10.2%) |
| Education level *n* (%) | |  |  |  |  |
|  | High school | 115 (16.5%) | 140 (29%) | 87 (23%) | 342 (21.9%) |
|  | Bachelor’s degree | 254 (36.4%) | 192 (39.8%) | 127 (33.6%) | 573 (36.7%) |
|  | Postgraduate degree (i.e., Master’s, Doctorate) | 329 (47.1%) | 150 (31.1%) | 164 (56.6%) | 643 (41.3%) |
